# Standalone portable xenon-129 hyperpolariser for multicentre clinical magnetic resonance imaging of the lungs

**DOI:** 10.1259/bjr.20210872

**Published:** 2022-01-31

**Authors:** Graham Norquay, Guilhem J Collier, Oliver I Rodgers, Andrew B Gill, Nicholas J Screaton, Jim Wild

**Affiliations:** 1 POLARIS, Department of Infection, Immunity & Cardiovascular Disease, University of Sheffield, Sheffield, UK; 2 Department of Radiology, Papworth Hospital NHS Foundation Trust, Cambridge, UK

## Abstract

**Objectives:**

Design and build a portable xenon-129 (^129^Xe) hyperpolariser for clinically accessible ^129^Xe lung MRI.

**Methods:**

The polariser system consists of six main functional components: (i) a laser diode array and optics; (ii) a B_0_ coil assembly; (iii) an oven containing an optical cell; (iv) NMR and optical spectrometers; (v) a gas-handling manifold; and (vi) a cryostat within a permanent magnet. All components run without external utilities such as compressed air or three-phase electricity, and require just three mains sockets for operation. The system can be manually transported in a lightweight van and rapidly installed on a small estates footprint in a hospital setting.

**Results:**

The polariser routinely provides polarised ^129^Xe for routine clinical lung MRI. To test the concept of portability and rapid deployment, it was transported 200 km, installed at a hospital with no previous experience with the technology and ^129^Xe MR images of a diagnostic quality were acquired the day after system transport and installation.

**Conclusion:**

This portable ^129^Xe hyperpolariser system could form the basis of a cost-effective platform for wider clinical dissemination and multicentre evaluation of ^129^Xe lung MR imaging.

**Advances in knowledge:**

Our work successfully demonstrates the feasibility of multicentre clinical ^129^Xe MRI with a portable hyperpolariser system.

## Introduction

Magnetic resonance imaging (MRI) of hyperpolarised xenon-129 (^129^Xe) is an emerging research and clinical imaging modality, with demonstrated clinical impact in a range of lung pathologies such as cystic fibrosis, interstitial lung diseases, asthma and obstructive lung diseases.^1^ Additionally, hyperpolarised ^129^Xe MRI has been demonstrated in other organs (e*.*g*.* the brain and kidneys) where dissolved-phase xenon can be used to study tissue perfusion and gas exchange.^2,3^ While conventional proton (^1^H) MRI makes use of proton spin densities in tissues and blood in the lungs, owing to the comparatively lower density of ^129^Xe in the lung airspaces or dissolved in blood and other tissues, hyperpolarised ^129^Xe MRI relies on exogenous enhancement (“hyperpolarisation”) of the magnetisation of the ^129^Xe nuclei using laser polarisation techniques.^4^ This enhancement leads to a five orders of magnitude MR signal enhancement for hyperpolarised ^129^Xe when compared to thermally polarised^129^Xe. This is critical in enabling MR images of ^129^Xe in the lung airspaces, as well as in well-perfused organs such as the kidneys^3^ and the brain.^5,6^ Early development of noble gas MRI of human lungs focussed on polarised helium-3 (^3^He) because of its isotopic purity and higher intrinsic MR signal.^7^ In recent years, however, noble gas lung MRI research has moved from ^3^He towards ^129^Xe due to reasons of greater availability and thus lower cost of xenon, which can be extracted from the air.^8^

For clinical applications of hyperpolarised ^129^Xe MRI, it is essential that xenon doses are generated rapidly (scan run time~10 min) with sufficient ^129^Xe polarisation levels (>20%) to enable clinical interpretation of ^129^Xe MR images.

The most common technique used to magnetise ^129^Xe is spin-exchange optical pumping.^4^ Two methods of spin-exchange optical pumping have emerged from physics research for clinical ^129^Xe MRI applications: batch mode^9,10^ and continuous-flow mode.^11,12^ The main operational difference between batch and continuous-flow mode is that batch mode does not require cryogenic accumulation of xenon, whereas continuous-flow mode does. This difference generally leads to two distinct performance regimes for batch and continuous-flow mode, namely (i) batch mode yields higher ^129^Xe polarisation (50%–90%) with slower xenon production rates (100–1000 mL/h) and (ii) continuous-flow mode yields lower ^129^Xe polarisation (20%–50%) but with faster production rates (1000–3500 mL/h). The faster production rates currently obtainable on continuous-flow hyperpolarisers offer increased potential for routine clinical ^129^Xe MRI when compared to batch mode systems given typical xenon throughput demands. For example, at the University of Sheffield, where the modality is being used clinically for lung disease assessment, the throughput is as many as five patients/day, each requiring between 1,000 mL and 3,500 mL of hyperpolarised ^129^Xe for a given MR scan protocol. While modern continuous-flow polarisers demonstrate the feasibility of clinical ^129^Xe MRI, widespread clinical uptake is still restricted by the need for additional lab space and/or the electrical and mechanical estates utilities necessary to install, house and run a commercially-available ^129^Xe polariser.

Our solution was to design and build a portable ^129^Xe polariser for rapid low-cost deployment for clinical ^129^Xe lung MRI. The design is a self-contained, stand-alone and transportable device that can quickly be installed on-site or be taken temporarily to another facility where there is interest in using hyperpolarised ^129^Xe. Thus removing the need for costly stationary equipment installation and not interfering with existing infrastructure required for operation. In this work we build on previous work aimed at increasing portability of ^129^Xe polariser technology,^13,14^ by developing a compact ^129^Xe hyperpolariser optimised for high-volume xenon production for clinical lung MRI in a hospital setting. The design criteria of the system were as follows: (i) readily transportable to an external clinical MRI facility; (ii) compact with no need for additional site infrastructure (i*.*e*.* it should run on mains electricity without the need for compressed air supplies), (iii) regulatory approved for in vivo use; and (iv) provide doses of ^129^Xe for high-quality clinical lung MRI in <20 min.

## Methods and materials

### Equipment

**Figure 1.**
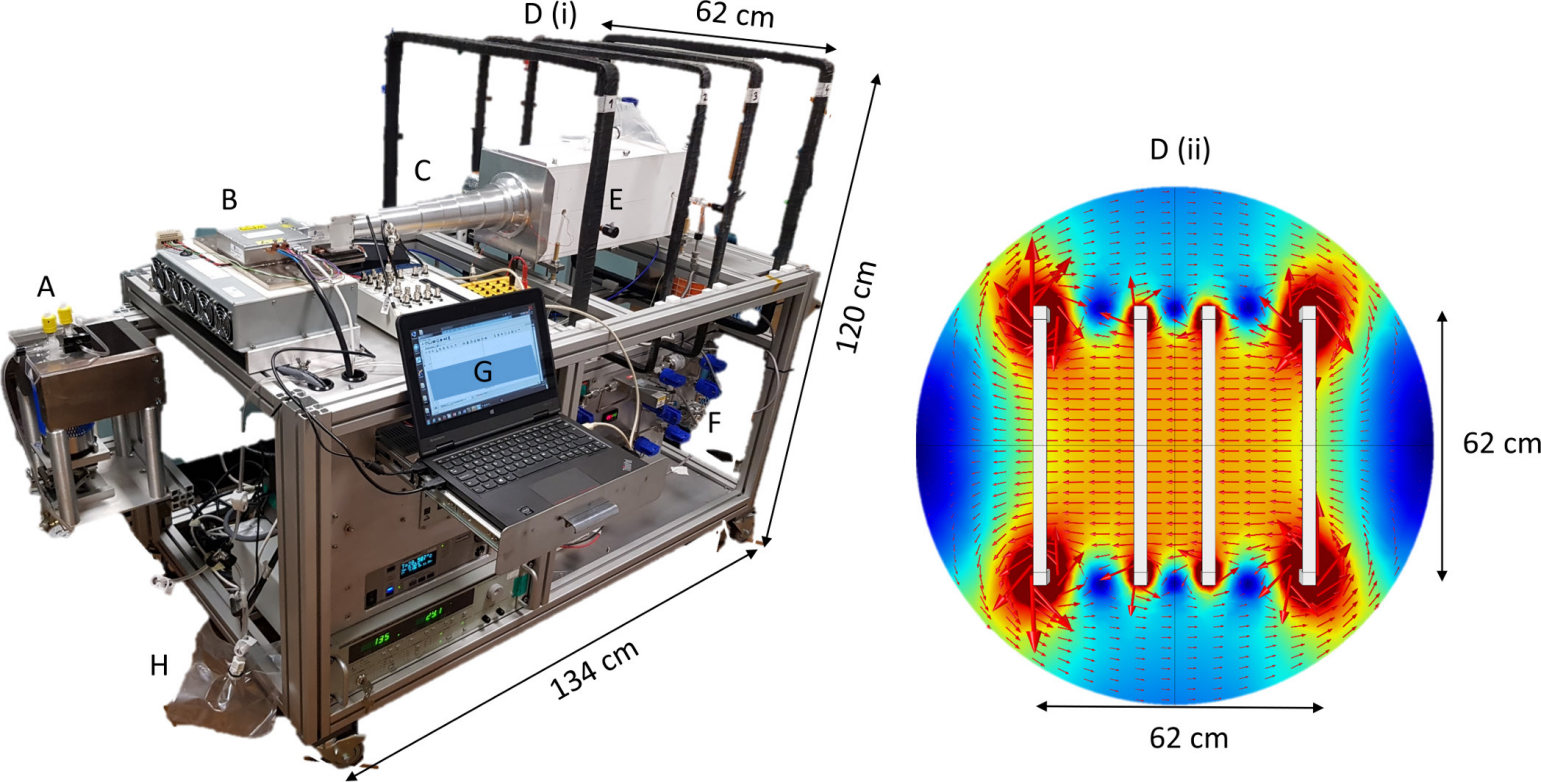
Photo of the assembled ^129^Xe polariser on the left and on the right a map of the 4-square coil geometry magnetic field coils. A is a retractable permanent magnet housing spiral glassware and an aluminium dewar for cryogenic accumulation of xenon. B is a 75W air-cooled laser diode and C is an optical train containing polarisation optics and a beam expander to output a 7.5-cm-diameter beam of circularly polarised light. D (i) and (ii) show the magnetic field coil placement and a simulation of the generated field lines (COMSOL Multiphysics). E is a ceramic forced-air oven housing a 40-cm-length cylindrical cell containing ~1 g droplet of rubidium metal. G is a computer workstation for on-board NMR of ^129^Xe in the cell and monitoring of the laser diode output. F shows the gas-handling manifold and H is a Tedlar bag for collection of sublimated hyperpolarised ^129^Xe gas.

The polariser comprises the following components: (i) a laser diode array with beam shaping optics; (ii) an electromagnetic static *B*_0_ coil assembly; (iii) an oven containing a cylindrical optical cell; (iv) NMR and optical spectrometers; (v) a laser power meter; (vi) a gas-handling manifold; and (vii) a cryostat within a permanent magnetic field. See Figure 1 for a photo of the polariser with labelled components. The laser light for optical pumping is generated by an air-cooled laser diode array (Brightlock Ultra-100^®^, QPC Lasers, Sylmar, US) providing ~50 W of laser light tuned to the rubidium valence electron *D*_1_ line (795 nm). The class IV laser diode is coupled to an optical fibre to custom optics that produce left circularly polarised light in a homogeneous circular beam that is incident on the oven window. Stray laser light is mitigated by enclosing the beam, optics and oven with front and back plates, however laser safety goggles are worn as an additional precautionary measure.

The oven is made of compressed calcium silicate ceramic with antireflection glass windows and a cylindrical optical cell made from borosilicate glass with volume 1767 cm^3^ (40 cm length, 7.5 cm diameter) loaded with ~1 g rubidium. The oven is heated using a bespoke air compressor / heating element combination. The oven and cell are contained within a magnetic field generated by a four-coil electromagnet using square coils with a side-length of ~60 cm for optimised field homogeneity [Figure 1, D (ii)]. The nominal *B*_0_ field is maintained at 2.4 mT, corresponding to a 28 kHz resonance frequency for ^129^Xe nuclei. The gas manifold directs gas flow from a cylinder (located in a gas trolley ~1 m away from the system to prevent magnetic field distortion) containing 3% isotopically enriched xenon (86% ^129^Xe), 10% N_2_ and 87% He (~2000 L volume at 2000 psi pressure, BOC, Linde gases group). After passing through a gas getter and pressure regulator set for a cell pressure of 2 bar, the gas enters the optical cell via high-vacuum PTFE stop-cock valves.

During continuous-flow operation, the xenon gas mixture is flowed through the cell in a direction counter to the incident laser light at a rate of 1000 sccm (standard cubic cm per minute), corresponding to a xenon flow rate of 1800 mL/h. The polarised gas exiting the cell is flowed through Tygon (Saint-Gobain, Coventry, UK) tubing towards spiral glassware. The glassware is held within a field of 250 mT from a NdBFe horseshoe permanent magnet and submerged in a dewar containing liquid N_2_, whereupon the xenon in the flowing gas mixture is cryogenically separated from He and N_2_, which are removed as exhaust gases through a vacuum line. The gas flow rate is controlled using a mass flow meter in line with a diaphragm vacuum pump which generates a downstream pressure of ~2 mbar. Once sufficient xenon snow is deposited in the spiral, it is defrosted by immersion in room-temperature water where the frozen xenon sublimates to the gas phase and flows into a Tedlar bag (Jensen Inert Products, Coral Springs, US) ready for QC release to the subject. In situ laser monitoring is provided by an optical spectrometer and a photon power meter. A home-built low-field nuclear magnetic resonance spectrometer using a LabVIEW software interface and a National Instruments data acquisition board (PCIe-6351 X-series multifunction DAQ) is used for in-system signal monitoring of the polarised ^129^Xe. The signals from ^129^Xe is acquired using a 100-turn surface *B*_1_ coil located on top of the optical cell inside the oven.

The assembled ^129^Xe polariser (see photo in Figure 1) is standalone, occupies a footprint of 1.34m length x 0.72m width x 1.2m height, is powered by 3 × 240 V / 50 Hz AC mains sockets, weighs less than 150 kg and can be moved easily by two people into a lightweight van. The polariser and its associated GMP processes have been regulatory approved for the manufacture of polarised ^129^Xe for clinical lung MRI by the UK Medicines and Healthcare products Regulatory Agency (MHRA) under the Manufacturing Specials licence number MS-18739.

### Lung imaging

**Figure 2.**
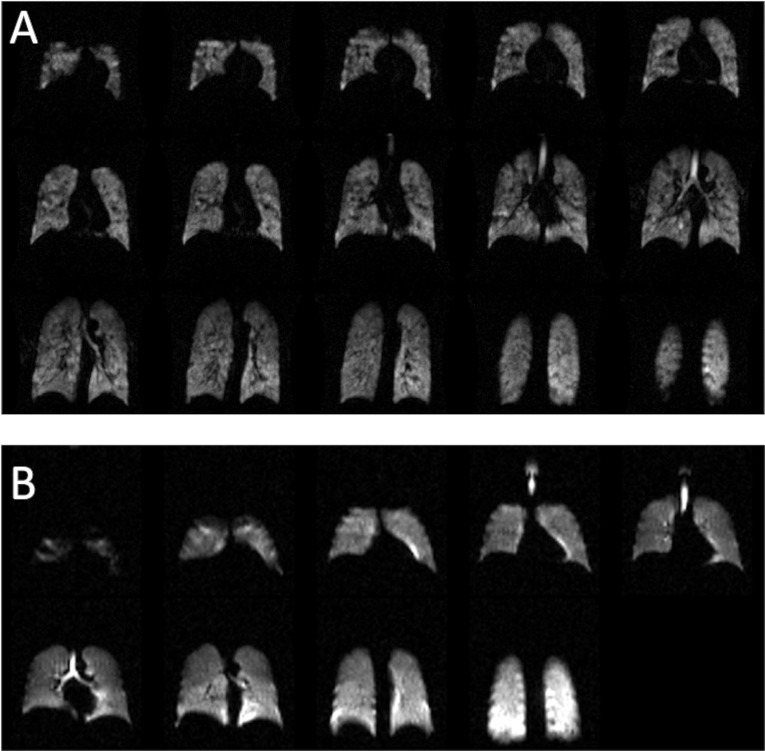
A: ^129^Xe lung ventilation MR images acquired on a 1.5 T clinical scanner at our imaging centre from a subject with asthma. Sequence details: repetition time msec/echo time msec, 6.7/2.3; field of view, 400×400 mm; matrix size, 80×80; slice thickness, 10 mm; flip angle, 10°; and bandwidth, 200 Hz/pixel. B: ^129^Xe lung ventilation MR images acquired on a 1.5 T clinical scanner at an external imaging centre the day after polariser transportation. Sequence details: volunteer, healthy male; repetition time msec/echo time msec, 6.9/2.3; field of view, 367×420 mm; matrix size, 56×64; slice thickness, 22 mm; flip angle, 10°; and bandwidth, 200 Hz/pixel.

To test the concept of portability of the polariser, it was transported 200 km in a van to a novice clinical radiology site with no previous in-house experience of ^129^Xe MRI. Lung imaging was performed using a transmit-receive vest coil (CMRS, US) on a healthy male volunteer the next day after 3 h of commissioning on site on a 1.5 T MR scanner (Siemens, MAGNETOM, Avanto). Prior to running the ventilation sequence, we calibrated the flip angle and centre frequency from an inhaled dose of 1L of a 3% xenon gas mixture. Sequence details are shown within the caption of Figure 2. The lung imaging work was conducted with full UK ethics approval (STH 20825, IRAS ref 265997) and UK regulatory manufacturing licence for xenon (MS-18739).

## Results

Figure 2A shows MR images of ^129^Xe acquired from the lungs of patients with the polariser in routine use at our MR imaging centre; inhaled dose 500 mL of xenon topped up to 1 litre with medical grade nitrogen acquired on a GE 450W 1.5 T MRI scanner housed in a relocatable mobile unit. Figure 2B shows ^129^Xe lung images from the healthy volunteer at the external imaging centre; inhaled dose 300 mL of xenon topped up to 1 litre with medical grade nitrogen. The ^129^Xe polarisation was measured to be ~30% (using the method described in ref.^15^), and the 300 mL and 500 mL doses were generated in 10 min and 17 min (xenon production rate of 1800 mL/h). The signal-to-noise ratio over all image slices was measured to be >40.

## Discussion

We have demonstrated the feasibility of a lightweight, high-volume production rate, portable ^129^Xe polariser for hyperpolarised gas lung MRI imaging. Portability of the system was demonstrated with a rapid turnkey installation at an external clinical imaging centre. From an installation and running perspective, the system fits easily in the back of a transit van, operates reliably on three 240V/50 Hz mains electricity sockets, and the on-board air compressor and heater is able to heat the oven to 140°C in ~20 min with <1°C fluctuation once thermal stability is reached. The system has been tested in various locations including in the close confines and noisy electrical environment of the equipment room of a relocatable mobile MRI scanner. The only additional requirements at the installation site are completion of risk assessments and also access to liquid nitrogen; this should not pose an impediment with respect to accessibility to other clinical MR centres, as all large hospitals should have access to liquid nitrogen storage.

In order to compare operational performance against other polariser systems, we employ the metric dose equivalence rate (DER),^16^ a metric that couples the quality and quantity of xenon gas available for patient inhalation within a given time period – i*.*e. it is a product of the operational ^129^Xe polarisation and the xenon volume production rate. It is quantified numerically as DER = f×P×Q, where f is the isotopic fraction of ^129^Xe (normalised to 1.00 in the calculation), P is the fractional ^129^Xe polarisation and Q is the xenon volume production rate.^16^ Using this formula with a Q of 1800 mL/h and P of 0.30 for the system described here yields a DER equal to 540 mL/h, which enables acquisition of diagnostic-quality MR lung images using 500 mL of xenon gas produced in <20 min. The performance of our portable polariser is approaching that of a permanent clinical ^129^Xe polariser system, which has a DER value of ~1000 mL/h and is used routinely for diagnostic clinical lung imaging.^12^ It is worth noting that to facilitate multicentre applications of ^129^Xe MRI, as well as increasing a site’s accessibility to polariser technology, it is important to standardise xenon dosing and imaging protocols as outlined in ref.^17^ and to also consider the additional outlay on ^129^Xe RF coils and broadband RF amplifiers on the scanner itself.

## Conclusions

In this work, we have demonstrated a self-contained, portable polariser that rapidly generates highly polarised ^129^Xe gas. The system routinely provides high-SNR lung imaging, and can easily be transported and installed at novice clinical imaging centres where no specialist equipment is required for polariser installation. The system should form the basis of a cost-effective platform for wider dissemination and multicentre clinical evaluation of hyperpolarised ^129^Xe MR imaging. Increased access to ^129^Xe hyperpolarisation technology outlined in this work should create new opportunities to explore and utilise the clinical impact of ^129^Xe MRI for diagnosis, prognosis, and treatment response monitoring of a number of diseases within the lungs and beyond.
